# Synthesis Method and Thermodynamic Characteristics of Anode Material Li_3_FeN_2_ for Application in Lithium-Ion Batteries

**DOI:** 10.3390/ma14247562

**Published:** 2021-12-09

**Authors:** Anatoliy Popovich, Pavel Novikov, Qingsheng Wang, Konstantin Pushnitsa, Daniil Aleksandrov

**Affiliations:** 1 Institute of Machinery, Materials, and Transport, Peter the Great St. Petersburg Polytechnic University, 195251 Saint Petersburg, Russia; director@immet.spbstu.ru (A.P.); novikov_pa@spbstu.ru (P.N.); pushnitsa_ka@spbstu.ru (K.P.); 2 CHN/RUS New Energy and Material Technology Research Institute, Huzhou 313100, China; envbattery@yandex.ru

**Keywords:** lithium-ion battery, anode battery, lithium-ion thermodynamics, solid-state synthesis

## Abstract

Li_3_FeN_2_ material was synthesized by the two-step solid-state method from Li_3_N (adiabatic camera) and FeN_2_ (tube furnace) powders. Phase investigation of Li_3_N, FeN_2_, and Li_3_FeN_2_ was carried out. The discharge capacity of Li_3_FeN_2_ is 343 mAh g^−1^, which is about 44.7% of the theoretic capacity. The ternary nitride Li_3_FeN_2_ molar heat capacity is calculated using the formula *C_p,m_* = 77.831 *+* 0.130 × T − 6289 × T^−2^, (T is absolute temperature, temperature range is 298–900 K, pressure is constant). The thermodynamic characteristics of Li_3_FeN_2_ have the following values: entropy S^0^_298_ = 116.2 J mol^−1^ K^−1^, molar enthalpy of dissolution Δ_d_H_LFN_ = −206.537 ± 2.8 kJ mol^−1^, the standard enthalpy of formation Δ_f_H^0^ = −291.331 ± 5.7 kJ mol^−1^, entropy S^0^_298_ = 113.2 J mol^−1^ K^−1^ (Neumann–Kopp rule) and 116.2 J mol^−1^ K^−1^ (W. Herz rule), the standard Gibbs free energy of formation Δ_f_G^0^_298_ = −276.7 kJ mol^−1^.

## 1. Introduction

In the world of technological development, energy sources are being severely depleted. In this regard, the issues related to creating new energy sources, in particular renewable energy sources, are being considered.

Secondary batteries, such as lithium-ion, lithium sulfur, and hydrogen batteries, are attracting increased attention for their development and production. Probably, one of the prospective renewable sources of energy is the lithium-ion battery (LIB) as an energy source for many applications, such as electric cars and buses, laptops, mobile phones, etc. LIBs solve the problems of high energy requirements (energy and power density, cycle life), environmental efforts, and relatively low cost.

A lot of efforts were directed to the development of more advanced batteries. For example, different approaches for LIB’s development were used, such as nanostructured materials [1,2,3,4,5,6,7,8,9,10,11,12,13], the growth of the capacity and voltage of cathode materials [14,15,16,17,18,19,20,21,22,23,24,25,26,27,28,29,30], hollow and porous and structures [13,31,32,33,34,35,36,37,38,39,40,41,42,43,44], safety issues, including separator and liquid electrolyte studies [45,46,47,48,49,50,51,52,53,54,55,56], etc. As a prospective current source for electric vehicles (EV), LIBs have proven their market position. To receive high-performance lithium-ion batteries, it is required to improve the specific capacity of active (electrode) materials. 

Thus, a lot of efforts were focused on the fabrication of anode materials with high theoretical specific capacity. For example, silicon has attracted the attention of the LIBs industry as an anode material with ultrahigh specific capacity (4212 mAhg^−1^), although the large volume expansion of silicon during the charge/discharge process (300%) leads to a capacity decrease and reduced cycle life [57]. 

Another popular anode material with high performance is Li_4_Ti_5_O_12_. This anode material attracted attention due to its low manufacturing cost, high safety, and environmental friendliness [58,59]. However, Li_4_Ti_5_O_12_ has poor electrical conductivity of 10^−8^–10^˗13^ S cm^−1^, a low lithium diffusion coefficient (10^−9^–10^−16^ cm^−2^ s^−1^), and a low theoretical capacity of 175 mAh g^−1^ [60,61,62,63]. 

Previous works shows good electrochemical properties of Li_3_N-type anodes, e.g., Li_2_Na_4_N_2_ and Li_4_Na_2_N_2_ phases [64], LiBeN [65], Li_3_N-Mg_3_N_2_ [66], Li_2n-1_*M*N [67], and Li_3_FeN_2_ [68,69]. Thus, as Li_3_FeN_2_ materials have transition metal, it could not be used as solid electrolyte because transition metals might produce conduction electrons, which is unacceptable for a solid electrolyte of lithium battery. However, this quality is advantageous for using this material as an electrode. Li_3-x_FeN_2_ (0 < x < 1) has a high capacity of 260 mAh g^−1^ [67]. In addition, the charge–discharge potentials between 0 and 2 V (vs. Li) were very flat for x = 0.1–0.7. 

Li_3_FeN_2_ was first synthesized by Frankenburger et al. by the reaction of lithium nitride (Li_3_N) with elemental Fe in N_2_ atmosphere [70]. After decades, Fromont investigated the reaction of Li_3_N with iron using thermogravimetry [71]. These studies show that Li_3_FeN_2_ was indexed by an orthorhombic cell with lattice parameters a = 9.65 Å, b = 8.66 Å, and c = 8.38 Å. Emery et al. [70] show the solid-state synthesis of Li_3_N with Fe powder in atmosphere, which shows a cationic mixing in Li_3_FeN_2_ compound. 

Li_3_FeN_2_ is a prospective material for hydrogen storage because of its hydrogen uptake capacity of 2.7 wt %, of which about 1.5 wt % was reversible [69,72,73].

In this article, the two-step synthesis and properties of promising anode material Li_3_FeN_2_ are shown. Firstly, Li_3_N synthesis was obtained in an adiabatic chamber. Then, mixed with iron nanopowder, Li_3_FeN_2_ was obtained at a tube furnace. Two-step synthesis was chosen for the synthesis of high-purity complex nitride Li_3_FeN_2_.

## 2. Materials and Methods

**Figure 1:**
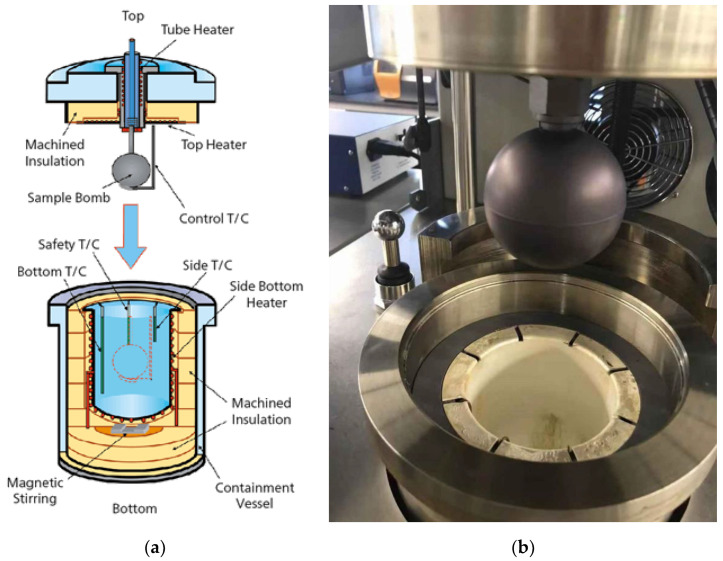
Scheme (**a**) and photo (**b**) of Netzsch APTAC chamber. 1—machined insulation; 2—sample bomb; 3—safety thermocouple; 4—bottom thermocouple; 5—magnetic stirring; 6—containment vessel; 7—machined insulation; 8—side bottom heater; 9—side thermocouple; 10—control thermocouple; 11—top heater; 12—tube heater.

A 16 mm diameter and 0.6 mm lithium plate sliced and polished in an argon glovebox, iron nanopowder, nitrogen, and ammonia (NH_3_) were used as starting components for Li_3_N, Fe_2_N, and Li_3_FeN_2_ synthesis (Table 1). The purity of materials shown in Table 1 is according to suppliers’ data. Lithium sliced plates were put into a titanic autoclave nitrogen-filled bomb of Netzsch APTAC 264 (Selb, Germany), as shown in Figure 1. The Li_3_N synthesis parameters are next: the temperature is 170 °C, heat rate is 2 °C/min, synthesis time is 6 h, and nitrogen pressure is ≈709.3 kPa (7 atm). 

Iron nanopowder and nitrogen were used as a source for Fe_2_N. Ceramic crucible with initial powder was put into the tube furnace (BTF−1700C, (Hefei, China). The tube has been purged by ammonia (NH_3_) for 30 min before synthesis. Synthesis was carried out in NH_3_ atmosphere at 530 °C for 6 h with a heat rate of 8 °C/min. Mechanically mixed and powder was hot pressed for 2 h at 1100 °C. The received hot-pressed sample was heated in N_2_ atmosphere for 10 h at 700 °C (heat rate was 5 °C/min). After heat treatment, the sample was mechanically ground into ivory-colored powder.

XRD analysis was held with a Bruker D8 Advance (Karlsruhe, Germany) with a step of 0.02°. Structural parameters were refined by the Rietveld method using TOPAS5 software.

X-ray diffraction analysis (XRD) was used as the structure analysis method for the synthesized nitrides powders investigated. XRD analysis was performed with a Bruker D8 ADVANCE diffractometer with a vertical goniometer and Cu K_α_-radiation. The diffraction angles (2θ) are 5–100°, 10–80°, and 5–120° for Li_3_N, Fe_2_N, and Li_3_FeN_2_, respectively.

Calorimetric measurements were performed using a TAM IV Microcalorimeter (Shanghai, China) at 298 K with the cell volume of 20 mL. Aqueous solution of 1 mol dm^−3^ HCl was used for the calorimetric cell ampoule. The ampoule was broken when thermal equilibrium was established, and nitride powder began to dissolve in HCl solution. Thermo-EMF vs. time was registered during the dissolution process providing the heat dissolution curve. Integration of this curve gave the value of dissolution enthalpy.

## 3. Results

**Figure 2:**
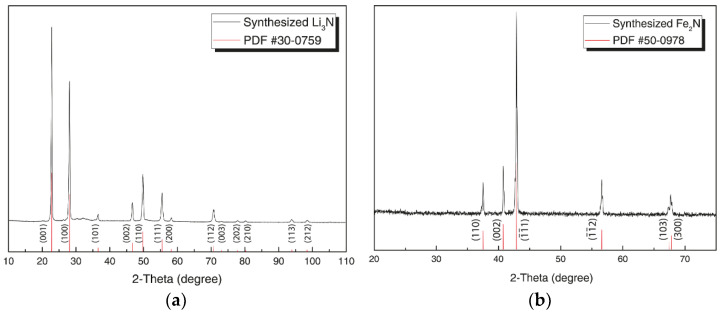
XRD pattern of synthesized (**a**) Li3N at 170 °C for 5 h at N2 atmosphere (709 kPa) and (**b**) Fe2N at 530 °C for 5 h at NH3 atmosphere.

Figure 2 shows the XRD pattern of synthesized Li_3_N (a) and Fe_2_N (b) powders. All peaks are in good correlation with database one. Li_3_N has a *P6/mmm* space group with lattice parameters a = 3.6711 Å, b = 3.6711 Å, and c = 3.8770 Å, which are in good correlation with [74] and PDF #30-0759. Fe_2_N reflection peaks also are in good correlation with [75] and PDF #50-0978. The space group of Fe_2_N is *P312* with lattice parameters a = 4.7912 Å, b = 4.7912 Å, and c = 4.416 Å.

**Figure 3:**
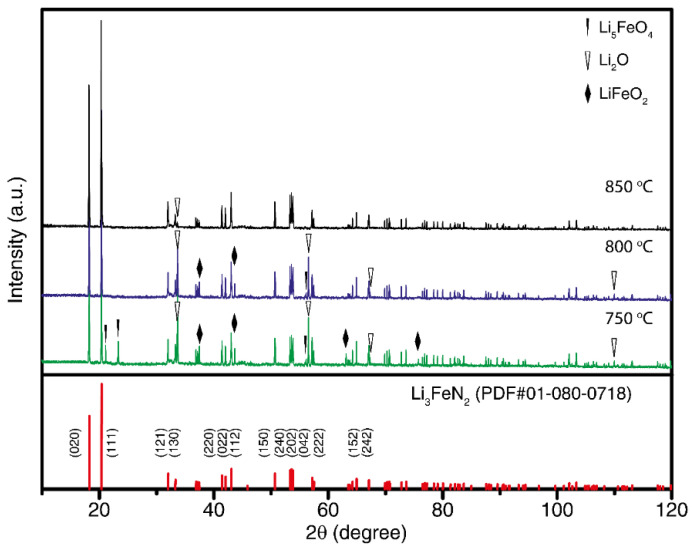
XRD patterns of Li_3_FeN_2_ after heat treatment at 750, 800, and 850 °C for 10 h in N_2_ atmosphere. The lines in the bottom indicate the diffraction positions of the Li_3_FeN_2_ structure (PDF #01-080-0718).

Figure 3 shows the XRD pattern of Li_3_N, Fe_2_N, and Li_3_FeN_2_ after heat treatment in an Netzsch APTAK chamber, tube furnace with ammonia atmosphere, and tube furnace with nitrogen atmosphere, respectively. Lattice parameters, *a* and *c*, calculated by the Rietveld method for Li_3_FeN_2_ are a = 4.872 Å, b = 9.677 Å, and c = 4.792 Å, respectively, in the *Ibam* space group. XRD patterns of Li_3_FeN_2_ synthesized at different temperatures are shown in Figure 3. The sample synthesized at 850 °C shows a high purity of 97.2% with Li_2_O impurity. Other samples include such impurities as Li_2_O (PDF #01-076-9237), Li_5_FeO_4_ (PDF #01-075-1253), and LiFeO_2_ (PDF #74-2284). Samples synthesized at 850 °C have only Li_2_O impurity; thus, further investigation of the compounds were conducted with materials synthesized at 850 °C.

The structure refinement defined that Li^+^ is in 4b and 8g, Fe^+3^ is in 4a, and N^−3^ is in 8j sites. All calculations were carried out with using TOPAS 4 software by Bruker. The final structure parameters (including site occupancy) are listed in Table 2. 

## 4. Discussion

### 4.1. The Standard Enthalpy of Formation

The formation enthalpy of Li_3_FeN_2_ (LFN) compound from single nitrides Li_3_N and Fe_2_N is calculated using the following equation (Δ_ox_H_LFN_):

Li_3_N + 0.5Fe_2_N + 0.25N_2_ → Li_3_FeN_2_, (1)

and single nitrides were synthesized by reactions, as described in the Experimental section:

6Li + N_2_ → 2Li_3_N, (2)

2Fe + 2NH_3_ → 2Fe_2_N + 3H_2_. (3)

**Figure 4:**
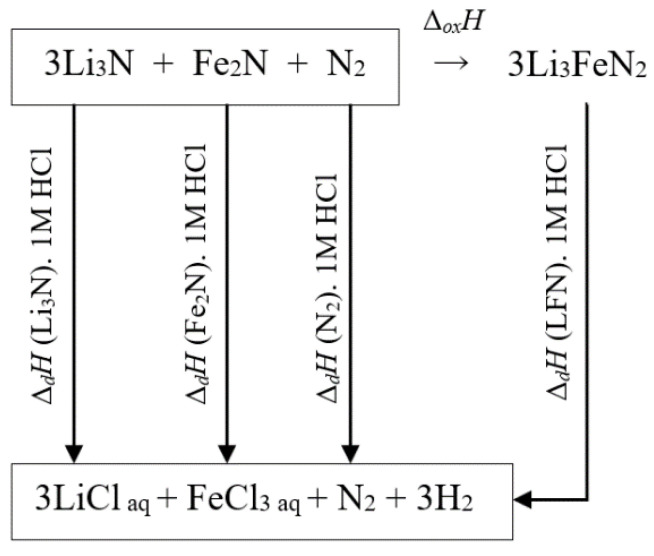
Thermochemical cycle scheme. Dissolution enthalpy connection of Li_3_FeN_2_ with its formation enthalpies from single nitrides.

For enthalpy calculation, we used thermodynamic cycle with the following reactions, as shown in Figure 4:

Li_3_FeN_2_ + 6HCl_(aq)_ → 3LiCl_(aq)_ + FeCl_3(aq)_ + N_2_↑+ 3H_2_, (4)

Li_3_N + 4HCl_(aq)_ → 3LiCl + NH_4_Cl, (5)

2Fe_2_N + 8HCl_(aq)_ → 4FeCl_2_ + 2NH_3_ + H_2_, (6)

N_2_ + 8HCl_(aq)_ → 2NH_4_Cl + 3Cl_2_, (7)

where (aq) means “aqueous”. The standard enthalpy (Δ_d_H_LFN_) has been determined in the calorimeter. The received value was equal to −1972.96 ± 25 J g^−1^, as shown in Table 3.

The resulting value of Δ_ox_H_LFN_ is obtained by the next equation:

Δ_ox_H_LFN_ = Δ_d_H_Li3N_ + 0.5Δ_d_H_Fe2N_ + 0.25Δ_d_H_N2_ − Δ_d_H_LFN_. (8)

The values of Δ_d_H_Li3N_, Δ_d_H_Fe2N_, and Δ_d_H_N2_ were also measured by the calorimetry method. Measurement results are shown in Table 3. The value of Δ_ox_H_LFN_ by Equation (8) is equal to −94.833 kJ mol^−1^. The negative value of Δ_ox_H_LFN_ defines Li_3_FeN_2_ as a stable phase. In addition, it is it is energetically favorable to synthesize LFN from single nitrides.

At last, the enthalpy of formation of Li_3_FeN_2_ from elements can now be calculated using the following equation:

Δ_f_H_LFN_ = Δ_f_H_Li3N_ + 0.5Δ_f_H_Fe2N_ + 0.25Δ_f_H_N2_ + Δ_ox_H_LFN_. (9)

Standard enthalpies for the calculation were taken from the handbooks [76,77], as shown in Table 4. 

The calculated value of the enthalpy of Li_3_FeN_2_ formation by Equation (9) is −291.331 ± 5.7 kJ mol^−1^, Table 4. The enthalpy of formation Δ_f_H^0^ for Li_3_FeN_2_ has the same order as for similar compounds, namely lithium metal nitrides (Table 4). That fact indirectly confirms the correctness of measurements. The value of formation enthalpy, calculated by Equation (9), can be used in thermodynamic estimation and the modeling of Li_3_FeN_2_ reactivity.

### 4.2. The Isobaric Heat Capacity

**Figure 5:**
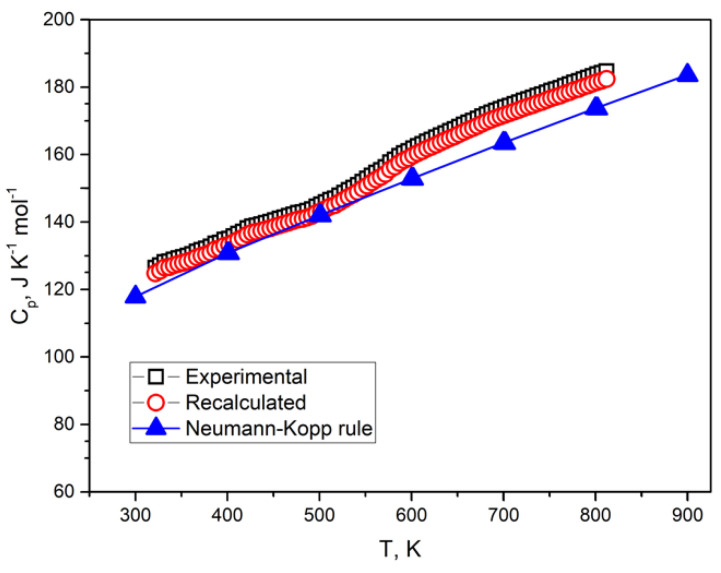
Temperature dependences of the experimental, recalculated, and Neumann–Kopp rule heat capacities of Li_3_FeN_2_. The line for the Neuman–Kopp rule is given as an approximating allometric line.

The temperature dependence of the isobaric heat capacity of the Li_3_FeN_2_ is shown in Figure 5. According to XRD data (Figure 3), the obtained powder material contains a certain amount of lithium oxide Li_2_O. This impurity quantity must be taken in consideration for valuation of the heat capacity of the Li_3_FeN_2_. This impurity could appear during the synthesis process or contact with oxygen in air atmosphere. XRD quantitative methods have limitations, but the heat capacity of a two-phase system must be recalculated by additive consideration:

mC_p_ = m(LFN)C_p_(LFN) + m(Li_2_O)C_p_(Li_2_O), (10)

where C_p_—a specific heat capacity (pressure is constant), and m—a mass. The sample weight consists of synthesized compound (Li_3_FeN_2_) and impurity (Li_2_O). So, the heat capacity of Li_3_FeN_2_) is expressed from Equation (10) as:

(11) $$C_{p}\left(\mathrm{LFN}\right)=\frac{{\mathrm{mC}}_{p}-m\left({\mathrm{Li}}_{2}O\right)C_{p}\left({\mathrm{Li}}_{2}O\right)}{m\left(\mathrm{LFN}\right)}.$$

The weight of the included compounds can be found from the total mass of the sample, which are calculated through the weight fraction of lithium oxide, ω(Li_2_O):

m(Li_2_O) = mω(Li_2_O) (12)

and

m(LFN) = m[1 − ω(Li_2_O)]. (13)

According to Equations (12) and (13), Equation (11) can be written as follows:

(14) $$C_{p}\left(\mathrm{LFN}\right)=\frac{C_{p}-C_{p}\left({\mathrm{Li}}_{2}O\right)\omega \left({\mathrm{Li}}_{2}O\right)}{1-\omega \left({\mathrm{Li}}_{2}O\right)}.$$

Thereby, the heat capacity of LFN can be calculated from the experimental data and heat capacity of lithium oxide impurity. For Equation (14), it is required to know the dependence of the specific heat capacity of the lithium oxide from temperature. For this, tabulated data for the lithium oxide heat capacity [77] were used. For the temperature range of 300–900 K, the commonly used polynomial formula for the heat capacity is as follows:

C_p_ = a + bT − cT^−2^ (15)

where a, b, and c are empirical coefficients; T is the absolute temperature. The received coefficients for lithium oxide are a = 76.666 J mol^−1^ K^−1^, b = –13.63·10^−3^ J mol^−1^ K^−2^, and c = –18.624·10^5^ J mol^−1^ K. The heat capacity of Li_3_FeN_2_ for the 300–900 K temperature range was recalculated using Equations (15) and (16) considering Li_2_O’s impurity presence. According to XRD data (Figure 3), Li_3_FeN_2_ contains about 2.8 ± 0.04 wt % Li_2_O. The experimental and recalculated LFN heat capacity is shown in Figure 5 and Table 5. Empirical values for heat capacity were calculated by the Neumann–Kopp rule. This rule prescribes calculating the molar heat capacity of a complex compound from the heat capacities of constituent elements by adding them in with the corresponding compound stoichiometry. However, this calculation method gives good results for room temperatures and rough results for high temperatures. For more accurate results, binary compounds were used instead of single elements:

(16) $$C_{p}\left(\mathrm{CN}\right)=\sum n\left(\mathrm{BN}\right)C_{p}\left(\mathrm{BN}\right)$$

where C_p_—molar heat capacity, n—a stoichiometric coefficient, and CN and BN are complex and binary nitrides, correspondingly. For LFN, Equation (16) can be written as (according to Equation (1)):

C_p_(LFN) = C_p_ (Li_3_N) + 0.5C_p_ (Fe_2_N) + 0.25C_p_ (N_2_). (17)

The dependence of the heat capacity by temperature calculated from Equation (17) using tabular data [77] is shown in Figure 5 and Table 5.

The temperature dependence of the heat capacity calculated by the Neumann–Kopp rule is in good correlation with the recalculated heat capacity (considering Li_2_O impurity amount). However, XRD quantitative analysis gives rough results for the small presence of compounds in the material. For other quantitative methods, the amount of impurities can be measured more accurately: for example, thermogravimetry or volumetric methods.

### 4.3. Entropy

Entropy is another thermodynamic function that should be calculated. The Third Law of thermodynamics states, “The entropy of a perfect crystal is zero when the temperature of the crystal is equal to absolute zero (0 K).”. Thus, the entropy absolute value can be valued by the equation:

(18) $$S\left(T\right)=\overset{T_{1}}{\underset{0}{\int }}\frac{C_{p}\left(T\right)}{T}\mathrm{dT}+\frac{\Delta H_{1}}{T_{1}}+\overset{T_{2}}{\underset{T_{1}}{\int }}\frac{C_{p}\left(T\right)}{T}\mathrm{dT}+\frac{\Delta H_{2}}{T_{2}}+\cdots +\overset{T}{\underset{T_{k}}{\int }}\frac{C_{p}\left(T\right)}{T}\mathrm{dT}$$

where S is entropy, ΔH_k_ is enthalpy of the k-th phase transition, and T_k_ is temperature of the k-th phase transition (0 < T_k_ < T). Since the entropy can be calculated by the Neumann–Kopp rule, if there is no phase transition until the calculation temperature, entropy can be also calculated by the Neumann–Kopp rule:

(19) $$S\left(T\right)=\overset{T}{\underset{0}{\int }}\frac{\sum C_{p}\left(T,\;\mathrm{BN}\right)}{T}\mathrm{dT}=\sum \overset{T}{\underset{0}{\int }}\frac{C_{p}\left(T,\;\mathrm{BN}\right)}{T}\mathrm{dT}=\sum S\left(T,\mathrm{BN}\right),$$

where BN is the binary nitride compound (see Equation (16)). According to Equations (16) and (17), Equation (19) can be written in the following way:

S (LFN) = S(Li_3_N) + 0.5S(Fe_2_N). (20)

The entropy of Li_3_FeN_2_ at room temperature is 113.2 J mol^−1^ K^−1^ according to Equation (20) and tabular data [82]. The additive rule for entropy calculation is suitable if the sum of the molar volumes of binary compounds differs a bit from the molar volume of the complex compound [83]. Thus, the molar volume for Li_3_N is 27.2 cm^3^ mol^−1^ (ρ = 1.28 g cm^−3^ [83]), for Fe_2_N is 19.8 cm^3^ mol^−1^ (ρ = 6.35 g cm^−3^ [83]), and for Li_3_FeN_2_ is 33.9 cm^3^ mol^−1^ (ρ = 3.09 g cm^−3^ [84]). The sum of the molar volumes of binary nitrides with their corresponding coefficients is 37.1 cm^3^ mol^−1^ and differs about 9% from the LFN molar volume, which allows usage of an additive scheme.

In addition, the LFN entropy can be calculated by the W. Herz rule [85]:

(21) $$S_{298}^{0}=K_{H}{\left(M/C_{p,298}\right)}^{1/3}m,$$

where K_H_ is Herz constant (K_H_ = 20.5), M is molar mass, C_p,298_ is isobaric heat capacity, and m is atoms per formula. According to Equation (21) and considering C_p,298_ from Table 5, the LFN entropy is 116.2 J mol^−1^ K^−1^. Thus, the LFN entropy calculated by the Herz rule is in good correlation with the Neumann–Kopp rule result.

### 4.4. The Standard Gibbs Free Energy

The enthalpy of formation and entropy calculated above allows evaluating the standard Gibbs free energy of Li_3_FeN_2_ formation (at T = 298 K):

(22) $$\Delta_{f}G_{298}^{0}=\Delta_{f}H_{298}^{0}-298\Delta_{f}S_{298}^{0}.$$

The resulting value of the Gibbs free energy for Li_3_FeN_2_ at room temperature is −276.7 kJ mol^−1^.

The next reaction is suggested for the determination of stability against metallic lithium with subsequent calculation of the Gibbs free energy at room temperature:

3Li + Li_3_FeN_2_ = 2Li_3_N + Fe. (23)

To determine the Gibbs free energy of the reaction, it is required to subtract from $\Delta_{f}G_{298}^{0}$ values of the Gibbs energy for initial reagents of the reaction. The $\Delta_{f}G_{298}^{0}$ for single elements is equal to zero, and for Li_3_N, it is −128.6 kJ mol^−1^ [82]. The Li_3_FeN_2_ Gibbs free energy has been calculated above. Thus, the Gibbs free energy for reaction (23) is 19.5 kJ mol^−1^, and this reaction is thermodynamically impossible. Finally, Li_3_FeN_2_ is stable against metallic lithium at room temperature.

## 5. Conclusions

The thermodynamic characteristics were determined for Li_3_FeN_2_ anode material for a lithium-ion battery. The two-step synthesis method allowed producing a highly pure compound with less than 3 wt % of Li_2_O impurity according to XRD data. The enthalpy of Li_3_FeN_2_ formation from binary nitrides was determined according to the measured enthalpy of dissolution of reagents and product of Li_3_FeN_2_ formation reaction. The obtained value is equal to −206.5 ± 2.8 kJ mol^−1^. The Li_3_FeN_2_ standard enthalpy of formation from single elements is equal to −291.3 ± 5.7 kJ mol^−1^. This value can be used in further thermodynamic modeling and determinations.

The heat capacity value was recalculated considering the presence of Li_2_O impurity. The temperature dependence of the heat capacity is in good correlation with calculation by the Neumann–Kopp rule. Finally, the heat capacity can be described by formula C_p_(T) = 78.997 + 0.132 × T + 4.654·10^5^ × T^−2^, where T is absolute temperature. The LFN entropy is equal to 113.2 J mol^−1^ K^−1^, and the Gibbs free energy of Li_3_FeN_2_ formation is −276.7 kJ mol^−1^. The calculations confirm that the Li_3_FeN_2_ material is stable against metallic lithium. All thermodynamic values and functions can be used for modeling and further calculations.

## Figures and Tables

**Table 1:** Summary of chemicals descriptions.

Name	Formula	Source	Purity, %
Iron nanopowder	Fe	Changsha Easchem Co., Ltd. (Changsha, China)	99.9
Lithium	Li	Xiamen Tmax Battery Equipments Ltd. (Xiamen, China)	99.9
Nitrogen	N_2_	Qingdao Guida Special Gas Co., Ltd. (Qingdao, China)	99.9–99.999
Ammonia	NH_3_	Wuhan Newradar Trade Company Ltd. (Wuhan, China)	99.9
Lithium nitride	Li_3_N	Prepared here	98.9 ^1^
Iron nitride	Fe_2_N	Prepared here	98.4 ^1^
Lithium iron nitride	Li_3_FeN_2_	Prepared here	99.1 ^1^

^1^ Purity according to XRD analysis.

**Table 2:** Structure characteristics of Li3FeN2.

Atom/Void	Site	g	x	y	z
Li_1_	8g	0.91	0.0	0.25745	0.25
Li_2_	4b	1	0.0	0.5	0.25
Fe	4a	1	0.0	0.0	0.25
N	8j	0.98	0.219979	0.113757	0.5

**Table 3:** Values of specific and molar enthalpies of dissolution (298 K, p = 101 kPa, 1 mol dm^−3^ HCl).

Compound	Specific Enthalpy, J g^−1^	Molar Mass, g mol^−1^	Molar Enthalpy of Dissolution, kJ mol^−1^	Ref.
Li_3_N	−3163.853 ± 30	34.83	−110.197 ± 1.7	this work
Fe_2_N	−13.79 ± 1.5	125.701	−1.734 ± 0.04	this work
N_2_	−71.716 ± 10	28.014	−2.56 ± 0.12	this work
Li_3_FeN_2_	−1972.96 ± 25	104.684	−206.537 ± 2.8	this work
Li_3_Na_3_N_2_	−2285.96 ± 13.4	117.807	−269.3018	[66]

**Table 4:** Standard enthalpies of formation from elements (Δ_f_H^0^).

Compound	Δ_f_H^0^_298_._15_, kJ mol^−1^	Reference
Li_3_N_(cryst)_	−196.78 ± 0.3	[76]
Fe_2_N_(cryst)_	−3.77 ± 0.1	[76]
N_2(gas)_	8.67 ± 0.1	[77]
Li_3_FeN_2(cryst)_	−291.331 ± 5.7	this work
LiCaN_(cryst)_	−216.8 ± 10.8	[78]
Li_3_BN_2(cryst)_	−534.5 ± 16.7	[79]
Li_3_AlN_2(cryst)_	−567.8 ± 12.4	[79]
LiMoN_2(cryst)_	−386.0 ± 6.4	[80]
Li_7_MnN_4_	−661	[81]

The subscripts (cryst) and (gas) mean “crystalline” and “gaseous”, correspondingly.

**Table 5:** The temperature dependence of the experimental (exp.), recalculated by Equation (14) (rec.), and calculated by the Neumann–Kopp (N-K) rule (Equation (17)) heat capacities (Cp) of Li_3_FeN_2_(s).

T, K	C_p_(exp.), J K^−1^ mol^−1^	C_p_(rec.), J K^−1^ mol^−1^	C_p_(N-K), J K^−1^ mol^−1^
300	126.9	124.1	117.8
400	134.1	132.6	130.7
500	146.3	144.3	141.9
600	160.5	158.3	152.8
700	173.8	171.9	163.4
800	183.3	180.7	173.6
900	186.1	178.8	183.5

## Data Availability

The data presented in this study are available on request from the corresponding author.
